# Genotype and environment interaction and stability of grain yield and oil content of rapeseed cultivars

**DOI:** 10.1002/fsn3.3023

**Published:** 2022-08-17

**Authors:** Seyed Hamed Qasemi, Khodadad Mostafavi, Mahmoud Khosroshahli, Mohammad Reza Bihamta, Hossein Ramshini

**Affiliations:** ^1^ Department of Biotechnology and Plant Breeding, Science and Research Branch Islamic Azad University Tehran Iran; ^2^ Department of Agronomy and Plant Breeding, Karaj Branch Islamic Azad University Karaj Iran; ^3^ Department of Biotechnology and Plant Breeding, Science and Research Branch Islamic Azad University Tehran Iran; ^4^ College of Agriculture & Natural Resources (UCAN) University of Tehran Karaj Iran; ^5^ College of Agriculture & Natural Resources University of Tehran Pakdasht Iran

**Keywords:** AMMI method, compatibility, GGE bi‐plot, Graphical analysis, stability

## Abstract

Investigating the interaction of genotype and environment in multi‐environment experiments (MET) is one of the reliable techniques to demonstrate the most stable and compatible cultivars. The main contribution of this study is to evaluate the stability and compatibility of rapeseed cultivars using additive main effects and multiplicative interaction (AMMI) and genotype plus genotype environment interaction (GGE) bi‐plot methods for grain yield and oil content. For this purpose, an experiment in a randomized complete block design (RCBD) with three replications was conducted for 10 rapeseed cultivars across 10 environments (five regions in 2 years). Hence, the proposed technique can be used to identify the superior cultivars corresponding to the multivariant properties including yield and oil content. To do so, a case‐study analysis was conducted over rapeseed, while more than 96% of the data variance for grain yield and more than 94% of the data variance for oil content were explained based on the AMMI model. According to the AMMI model, it was observed that the “Zarfam” and “Licord” genotypes were introduced as favorable genotypes for grain yield and oil content, respectively. “Karaj1” and “Sanandaj1” were selected as the superior environments for yield trait, “Kashmar2” for oil content, and “Licord” and “Kashmar2” were identified as the superior genotypes and environment for oil content, respectively. Graphical GGE bi‐plot illustrated that “Hyola401,” “Okapi,” and “Sarigol” for grain yield and “Option500” and “Sunday” for oil content were identified as stable and high‐yield genotypes. “Sanandaj1” for grain yield and “Karaj2” for oil content were identified as environments with high differentiation and screening power.

## Graphical abstract

This study was performed to evaluate the stability and compatibility of rapeseed cultivars in terms of grain yield and oil content in ten rapeseed cultivars in ten environments

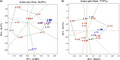

## 1 INTRODUCTION

Rapeseed is one of the most important oilseeds in the world, so the research about its cultivation and breeding of new varieties is essential (Friedt et al., 2018). The properties of rapeseed oil make it the healthiest and the most important edible vegetable oil (Loganes et al., 2016). In general, different cultivars with high genetic diversity are produced after breeding programs, so it is vital to identify cultivars with high adaptability in a wide range of environments (Mohammadi et al., 2014). One of the goals of plant breeding is to select cultivars with wide adaptation in a series of environments that usually have a proper and stable performance. The most appropriate method for identifying desirable cultivars with high productivity and extensive adaptation to most regions is multi‐environment experiments (MET) in target environments (Tena et al., 2019).

The genotype–environment interaction is challengeable for studying and measuring the performance and stability of cultivars in different environmental conditions. This is because it leads to a considerable variation in yield. This effect will be more significant when experiments are performed in different locations and years. The existence of genotype–environment interaction causes significant differences between the reactions of genotypes in different environments, which reduce the relationship between phenotypic appearance and genotypic values. The mismatch of phenotypic and genotypic values has caused the breeders to make an incomplete selection in phenotypic compatibility tests and not be able to evaluate performance accurately and identify the stable genotypes (Akbarpour et al., 2014; Neisse et al., 2018). Therefore, a multilocation experiment due to genotype interaction in the environment is suitable for identifying cultivars with suitable compatibility (Maniruzzaman et al., 2019). Various models have been developed to interpret genotype interactions across diverse environments. Parametric and nonparametric univariate methods were two desirable methods with relatively good efficiency and ease of use. However, these methods are inadequate to interpret the interaction of genotype × environment due to its complex and multidimensional nature (Ndhlela et al., 2014). Thus, to solve this problem, multivariate statistical methods are applied today. Among multivariate methods, additive main effects and multiplicative interaction (AMMI) and genotype plus genotype environment interaction (GGE) bi‐plot methods are widely used for this purpose (Neisse et al., 2018).

Analysis of variance (ANOVA) and principal component analysis (PCA) can measure the main additive effects and multiplicative interaction by using the AMMI model, respectively. The AMMI model is a combination of ANOVA and PCA with multiplicative parameters in a single analysis. In the AMMI model, to start with, the main effects of genotypes and environment are estimated with ANOVA (main effects). Then, the PCA evaluates the interaction of genotype with the environment (multiplicative interactions). The AMMI model fits the sum of several multiplicative terms rather than only one multiplicative term in assessing the performance of genotypes in different environments (Aduening et al., 2017; Bocianowskia et al., 2020). AMMI has several models: AMMI0, which estimates the main additive effect of genotypes and environments and does not include any major axis (IPCA). AMMI1, which combines AMMI0 genotype additive effects with environmental interactions estimated from the first principal component axis (IPCA 1); AMMI2, and so forth, until the full model with all IPCA axes (Nowosad et al., 2016). The GGE bi‐plot method is applied to visually evaluate the correlation of the studied traits through the Genotype × Trait bi‐plot diagram (Kaplan et al., 2017). The efficiency of this method in selecting genotypes with appropriate compatibility and stability in different products has been confirmed (Oliveira et al., 2018).

The GGE bi‐plot method offers a more efficient technique for analyzing the interaction of genotypes and environment because it can provide bi‐plots, in addition to a visual understanding of the interactions, while other methods of analysis such as Eberhart and Russell provide only information about genotype evaluation (Kaplan et al., 2017; Rezaizad et al., 2018). The data structure required by AMMI and GGE bi‐plot analyses is a two‐way matrix with the number of genotypes tested in several environments that combine the two statistical processes: ANOVA and PCA (Mohammadi et al., 2014). AMMI and GGE bi‐plot both reinforce each other for better and more reliable decision‐making, despite different approaches. These methods provide an accurate estimate of the interaction of a genotype in each environment and help identify suitable genotypes for specific environments (Erdemci, 2018; Neisse et al., 2018). Rezaizad et al. (2018), after evaluating 22 rapeseed genotypes, showed that the results of the AMMI analysis to identify stable genotypes are the same as those of the GGE bi‐plot analysis. Rahnejat and Farshadfar (2015) studied rapeseed genotypes in four different regions of Iran and identified “Okapi,” "Modena,” and “GKH 305” as high‐yielding and compatible genotypes (Rahnejat & Farshadfar, 2015).

The objective of this study includes comparing the two AMMI and GGE bi‐plot methods and identifying which one is more useful. Also, this study was planned to identify the most stable cultivars across environments. In this study, the superior cultivars in terms of grain yield and oil content in the studied environments were identified. Also, the environments with high differentiation power were found using AMMI and GGE bi‐plot methods.

## 2 MATERIALS AND METHODS

To compare the two methods, AMMI and GGE bi‐plot, and also to evaluate the stability and compatibility, an experiment in the form of a randomized complete block design (RCBD) for grain yield and oil content traits was performed with three replications in 10 different environments (five locations and 2 years) on 10 rapeseed cultivars. Experimental locations included Karaj, Birjand, Kashmar, Shiraz, and Sanandaj. Prior to the experiments, the locations were selected, based on the latitude, longitude, various climatic characteristics, and soil texture type. Karaj region has a longitude of ‘E54°50’ and a latitude of ‘N55°35’ with 1312 m above sea level and an average annual rainfall of 247.3 mm. Birjand region has a longitude of 32°52′N and a latitude of 59°12′E with 1491 m above sea level and an average annual rainfall of 171 mm. Kashmar region has a longitude of 58°29′59.99″E and a latitude of 35°14′60″N with 1063 m above sea level and an average annual rainfall of 166.5 mm. Shiraz region has a longitude of 29°32′N and a latitude of 52°36′E with 1484 m above sea level and an average annual rainfall of 324.2 mm. Sanandaj region has a longitude of 46°59′55.79″ and a latitude of 35°18′53.82″N with 1538 m above sea level and an average annual rainfall of 278 mm. The experimental plots consist of four rows with a distance of 0.5 m, length of 4 m, and distance between plants being 20 cm. All planting, holding, and harvesting operations were regularly and accurately performed. Sampling was accomplished to remove marginal effects from the two middle rows. Five plants were randomly taken into consideration to measure the traits. Accordingly, the average of all samples was calculated. Grain yield in terms of kg/ha and ton/ha was measured, after removing the margin effects. Tables 1 and Table 2 show the characteristics of genotypes and geographical specifications.

**TABLE 1:** Names and codes of canola cultivars studied in the experiment

Genotype No.	Genotype	Origin	Genotype No.	Genotype	Origin
G1	Sarigol	Iran	G6	Licord	Germany
G2	Hyola308	Canada	G7	Okapi	France
G3	Option500	Germany	G8	Hyola401	Canada
G4	Opera	Sweden	G9	Zarfam	Iran
G5	Sunday	Denmark	G10	Modena	Denmark

**TABLE 2:** Geographical specifications of the experiment performed areas

Area	Longitude	Latitude	Elevation AMSL (m)	Average rainfall (mm)
Karaj	50°54′E	35°55′N	1312	295.0
Birjand	59°12′E	32°52′N	1491	171.0
Shiraz	52°36′E	29°32′N	1484	337.8
Kashmar	58°48′E	35°53′N	1109	178.0
Sanandaj	47°00′E	35°20′N	1373	492.0

### 2.1 AMMI model

This model was introduced by Gauch (1992) and is an integrated model of ANOVA and PCA. First, the main effect of genotypes and environment was calculated by ANOVA techniques. Then, using the single‐value analysis technique (singular value decomposition), the genotypic and environmental components of the interaction were computed for the incremental deviation matrix (Crossa et al., 1991). Therefore, in the AMMI method, genotype interaction and the environment for the Yij data matrix are divided into two parts. One part includes the systematic structure of the interaction between genotype and environment, which is used to model and interpret the interaction of genotypes with the environments. The other part includes the residual (error) interpretable structure of the genotype and environment interaction (Farshadfar et al., 2011).

The AMMI model is presented:

(1) $$Y_{\mathrm{ij}}=\mu +g_{i}+e_{j}+\sum_{k=1}^{n}\lambda_{k}\alpha_{\mathrm{ik}}\gamma_{\mathrm{jk}}+e_{\mathrm{ij}}$$

*Y*_*ij*_: the yield of the *i* genotype in the *j* environment. *g*_*i*_: the mean of the i genotype. *λ*_*k*_: the square root of the eigenvalue of the PCA axis *k*, *α*_*ij*_. *ɣ*_*jk*_: the principal component scores for PCA axis *k* of the I genotype and the *j* environments, and *e*_*ij*_ is the residual.

Investigating the multiplicative effect of the matrix deviation from the additive effect (interaction matrix) can be obtained, as described in Equation 2 (Gauch et al., 2008).

(2) $$Z_{\mathrm{ij}}=Y_{\mathrm{ij}}–Y_{.j}–Y_{i.}+Y_{..}$$

There is an assessable multiplier effect component for an interaction matrix with the smallest dimension, which is usually the number of environments. It is challenging to interpret many interaction components; therefore, a method should be used to explain the maximum part of the variance of the interaction with the least possible components. The decomposition technique into single values is a suitable tool for this purpose.

### 2.2 GGE bi‐plot method

Graphical analysis was performed using the GGE bi‐plot method based on the analysis of individual values according to the following equation (Yan, 2012):

(3) $$Y_{\mathrm{ij}}-\mu -\beta_{j}=\lambda_{1}\;\xi_{i1}\;\eta_{j1}+\lambda_{2}\;\xi_{i2}\;\eta_{j2}+\varepsilon_{\mathrm{ij}}$$

where: $y_{\mathrm{ij}}$: Mean yield of genotype i in environment j. $\lambda_{2},\lambda_{1}$: Specific values for first principal component (PC1) and second principal component (PC2). $\xi_{i2},\xi_{i1}$: PC1 and PC2 scores for genotype *i*. $\;\eta_{i2},\eta_{i1}$: PC1 and PC2 scores for the environment *j*. $\varepsilon_{\mathrm{ij}}$: The residual of the model related to genotype *i* in environment *j*.

In other words, this method is a kind of PCA for the sum of the main effect of genotype and the interaction of genotype and environment. The procedure of decomposition into single values is used. Data obtained from genotypes and environments are analyzed as a two‐way matrix, and special values and specific vectors of genotypes and environments are extracted.

To display and scale PC1 and PC2 in a bi‐plot, the equation is rewritten as follows:

(4) $$y_{\mathrm{ij}}-{\bar{y}}_{j}=\xi_{i1}^{*}\eta_{j1}^{*}+\xi_{i2}^{*}\eta_{j2}^{*}+\varepsilon_{\mathrm{ij}}$$

In this equation, Code 2 and *n* = 1

$$\eta_{\text{in}}^{*}=\lambda_{n}^{1/2}\eta_{\text{in}},\xi_{\text{in}}^{*}=\lambda_{n}^{1/2}=\lambda_{n}^{1/2}\xi_{\text{in}}$$

The advantage of this measurement method is that PC1 and PC2 have the same unit.

After genotype‐specific vectors and bi‐plot symmetric scaling, GGE is obtained automatically. The software has completed all these steps and calculates and generates the required graphs.

Due to the existence of different units of traits, standardization of traits was used to eliminate units.

(5) $$Z=\frac{X-\mu }{\sigma }$$

In this equation, *Z*: standard score, *X*: initial data of the trait, *μ*: mean of the trait, and *σ*: standard deviation of the trait.

## 3 RESULTS AND DISCUSSION

### 3.1 Analysis of variance AMMI

The ANOVA of the AMMI model in grain yield in 10 rapeseed cultivars in 10 regions (five environments and 2 years) showed that the interaction between genotype and environment was significant. The coefficient of variation (CV) was small (19.57), indicating the experiment's accuracy in the study areas. Also, the effects of genotype and environment were significant on measured traits.

The interaction between genotype and environment indicates that the yield of genotypes differs from location to location, so the stability of grain yield can be studied. The significance of the effect of the environment also indicates that the environments differ in terms of genotype performance. Altogether, 90.99% of total variation was explained by PCA where 71.13% was related to first principal component (PC1) and 19.86% related to second principal component (PC2) (Table 3). Other researchers have also shown similar results in their multi‐environment experiments (MET) on other crops such as maize (Shiri & Bahrampour, 2015) and sunflower (Hemmati et al., 2018) that the environmental effect accounts for a significant percentage of the changes in data.

**TABLE 3:** Analysis of variance (ANOVA) by the additive main effects and multiplicative interaction (AMMI) method in grain yield in 10 rapeseed genotypes in 10 regions

Source	DF	SS	MS	F	*p*‐Value
Environment (E)	9	0.0006	0.000071	65.25	<.0001**
Replication	2	0.000003	0.0000018	1.7	.1850^ns^
Genotype (G)	9	0.0002	0.000029	26.95	<.0001**
Interaction (GE)	81	0.00013	0.000001	1.55	.0072**
PC1	17	0.000097	0.000005	5.26	<.000001**
PC2	15	0.00002	0.000001	1.66	<.006**
Pulled	49	0.000012	0.0000009		
Residual	198	0.00021	0.000001		
Total	299	0.0012			
CV%		19.57			

*, ** and ns: Significant at 5%, 1% and nonsignificant.

The component of the interaction effect of genotype × environment enables identifying a genotype with the viability and productivity in a particular environment. It means that a genotype may not show a similar performance when cultivated in another location. For this reason, after producing elite lines or hybrid genotypes, they should be tested in different environments (Torres Filho et al., 2017).

**FIGURE 1:**
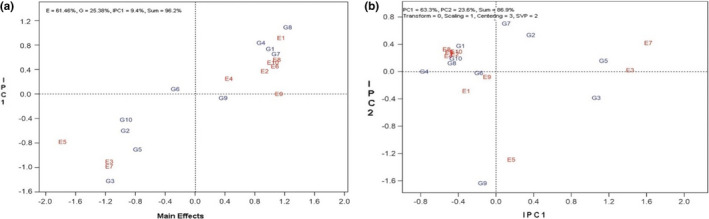
(a) AMMI1 (additive main effects and multiplicative interaction) diagram on grain yield in 10 rapeseed genotypes in 10 study areas. (b) AMMI2 diagram on grain yield in 10 rapeseed genotypes in 10 study areas

The table of the first and second components of the interaction showed that Zarfam, Modena, and Hyola308 genotypes had the lowest value for the first component, which showed the stability of these cultivars compared to other genotypes. Considering that the Zarfam genotype had a higher average yield (5.4 t/ha) than Modena and Hyola308, this cultivar was identified as having more stability and a higher relative average yield. In the study of environments, KARAJ1 and SANANDAJ1 environments had the highest amount of the first interaction component and were identified as environments with high screening power and differentiation. Also, the average yield in these locations was higher than those of other locations (Table 4). In the AMMI model, the x‐axis interprets genotypes and environment main effect, and the y‐axis represents the interactions between genotype with environment. Genotypes and environment showed much greater diversity in main and interaction effects. The environment above the y‐axis means it is desirable and shows high performance, while the environment below the y‐axis means it is undesirable and inefficient (Kendal et al., 2016). Based on the AMMI1 diagram obtained regarding grain yield traits in these cultivars, Hyola401, Opera, Sarigol, and Okapi genotypes were selected with the highest mean yield and relative stability. Also, Zarfam and Licord genotypes were identified as stable genotypes, compared to other cultivars due to their proximity to the origin of this chart (Figure 1a). In the AMMI2 diagram, which showed the performance of genotypes in specific environments, Sunday, Hyola308, and Okapi genotypes in SHIRAZ1 and BIRJAND1 regions, Option500 genotype in the KASHMAR1 region, Licord genotype in KARAJ1 and SANANDAJY1 and ZANDAJA1 regions, Modena and Opera in KARAJ2, BIRJAND2, KASHMAR2, and SANANDAJ2 regions had the highest yield. They were superior to other genotypes in these regions (Figure 1b).

**TABLE 4:** The amount of first and second major components in 10 rapeseed genotypes in 10 regions

Genotype	Yield Mean	IPC1	IPC2	Environment	Yield Mean	IPC1	IPC2
Sarigol	6	−0.022	−0.016	KARAJ1 (E1)	6.5	0.036	−0.0009
Hyola308	4.3	0.018	0.002	KARAJ2 (E2)	6	−0.009	−0.005
Option500	3.9	0.042	0.001	BIRJAND1 (E3)	3.9	−0.003	−0.005
Opera	6	−0.025	0.015	BIRJAND2 (E4)	5.4	−0.011	0.001
Sunday	4.6	0.027	0.006	KASHMAR1 (E5)	2	0.016	0.032
Licord	5.2	−0.002	0.001	KASHMAR2 (E6)	6.3	−0.01	0.001
Okapi	6.3	−0.019	−0.011	SHIRAZ1 (E7)	3.9	−0.004	−0.01
Hyola401	6.7	−0.033	0.003	SHIRAZ2 (E8)	6.3	−0.015	0.005
Zarfam	5.4	0.0018	0.033	SANANDAJ1 (E9)	6.4	0.02	−0.019
Modena	4.3	0.012	−0.034	SANANDAJ2 (E10)	6.2	−0.015	0.005

**FIGURE 2:**
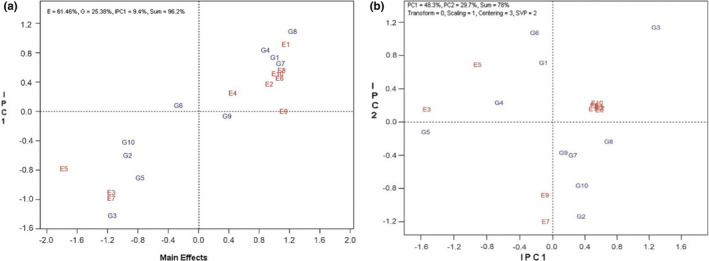
(a) AMMI1 (additive main effects and multiplicative interaction) diagram of oil percentage in 10 rapeseed genotypes in 10 study areas. (b) AMMI2 diagram of oil percentage in 10 rapeseed genotypes in 10 study areas

The ANOVA of the AMMI model in oil percentage also showed that the effects of genotype, environment, and interaction of genotype and environment were significantly different. The coefficient of variation (CV) was equal to 6.44%, which indicates the high accuracy of the experiment. In this analysis, the first two components explained 86.09% of the total variance of the interaction. PC1 and PC2 explained 68.49% and 17.59% of variation, respectively (Table 5). The results obtained from the table of the first and second components of the interaction also showed that Modena and Licord genotypes had the lowest value for the first component. Still, the Licord genotype had a higher percentage of oil (41%) than the Modena genotype (39%). In the study of test location, the KASHMAR2 region had the highest amount of the first component and also had the highest percentage of oil (47%), which was identified as an environment with high screening power of genotypes (Table 6). In the study of the AMMI1 diagram related to oil content, 94.3% of the total variance of the data was explained, of which 63.98% was related to environmental effect, 18.06% was related to genotype effect, and 12.3% was associated with the first interaction component. This diagram identified Option500, Hyola401, Hyola308, and Licord genotypes as high mean value. Among these genotypes, the Licord cultivar was identified as a more stable genotype as a result of its proximity to the origin of the diagram (Figure 2a). The AMMI2 diagram also explained 78% of the total variance of the data, of which 48.3% was related to PC1 and 29.7% was related to PC2. According to the AMMI2 diagram, Licord, Sarigol, and Opera genotypes in KASHMAR1 and BIRJAND1 regions and Option500 genotypes in KARAJ1, KARAJ2, BIRJAND2, KASHMAR2, SHIRAZ2, and SANANDAJ1 regions had the highest superiority (Figure 2b).

**TABLE 5:** Analysis of variance (ANOVA) by the additive main effects and multiplicative interaction (AMMI) method in oil percentage in 10 rapeseed genotypes in 10 regions

Source	DF	SS	MS	F	*p*‐Value
Environment (E)	9	0.0025	0.00028	40.86	<.0001**
Replication	2	0.000003	0.000001	0.28	.7569^ns^
Genotype (G)	9	0.0007	0.00007	11.53	<.0001**
Interaction (GE)	81	0.0007	0.000008	1.27	.0089**
PC1	17	0.00048	0.00002	4.15	<.000001**
PC2	15	0.00012	0.000008	1.21	.026*
Pulled	49	0.00009	0.000008		
Residual	198	0.001			
Total	299	0.005			
CV%		6.44			

*, ** and ns: Significant at 5%, 1% and nonsignificant.

**TABLE 6:** The amount of first and second major components in 10 rapeseed genotypes in 10 regions

Genotype	Oil% mean	IPC1	IPC2	Environment	Oil% mean	IPC1	IPC2
Sarigol	39	−0.0027	−0.022	KARAJ1 (E1)	40	−0.013	0.003
Hyola308	42	0.014	0.04	KARAJ2 (E2)	38	0.025	−0.003
Option500	43	0.078	−0.022	BIRJAND1 (E3)	37	−0.063	−0.037
Opera	40	−0.023	−0.019	BIRJAND2 (E4)	42	0.033	−0.004
Sunday	38	−0.066	−0.016	KASHMAR1 (E5)	38	−0.034	−0.029
Licord	41	0.01	−0.038	KASHMAR2 (E6)	47	0.04	−0.005
OKAPI	39	−0.004	0.017	SHIRAZ1 (E7)	38	−0.03	0.049
Hyola401	41	0.02	0.017	SHIRAZ2 (E8)	43	0.034	−0.005
Zarfam	38	−0.026	0.015	SANANDAJ1 (E9)	38	−0.026	0.039
Modena	39	0.0006	0.028	SANANDAJ2 (E10)	42	0.033	−0.004

Genotype environment interaction bi‐plot method effectively identified genotypes with compatibility, stability, and appropriate yield. Differences presented by genotypes for grain yield traits are the basis for proposing genotypes and expressing the possibility of selection for genetic improvement of species (Richardson & Paulo, 2020).

Mega Environment Identification: Visualizing the “which won where” pattern is vital for searching mega environments in different areas and evaluating experiment sites and genotypes in large environments (Yan et al., 2007).

**FIGURE 3:**
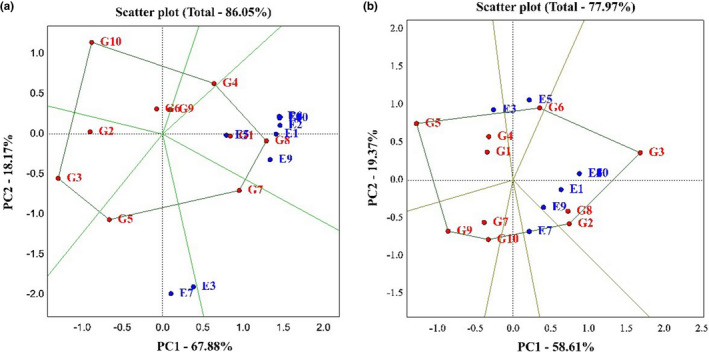
(a) Polygon views of the genotype plus genotype environment interaction (GGE) bi‐plot based on symmetrical scaling for the which‐won‐where pattern of genotypes and environments for yield trait. (b) Polygon views of the genotype plus genotype environment interaction (GGE) bi‐plot based on symmetrical scaling for the which‐won‐where pattern of genotypes and environments for oil percentage trait.

Based on the polygon diagram obtained from the GGE bi‐plot method on grain yield, the first component explained 67.88% and the second component 18.1% and a total of 86.05% of the total variance of the data. The different genotype's performance and their interaction with several environments are obtained using the GGE bi‐plot analysis by PC1 and PC2 factors (Arshadi et al., 2018). According to this diagram, which is obtained by connecting the genotypes that are farthest from the origin and the experiment environments, they are divided into mega environments by lines perpendicular to these polygons. Modena, Opera, Hyola401, Okapi, Hyola308, and Option500 genotypes were at the top of this polygon and were identified as desirable genotypes. Also, 10 studied environments formed three mega environments. The first mega environment included the SHIRAZ1 environment, the second mega environment included the BIRJAND1 environment, and other locations were placed in the third mega environment. As this diagram shows, the Hyola401 genotype had a higher yield in most environments than the different genotypes. The Licord and the Zarfam genotypes did not react to environmental changes due to their vicinity to the diagram origin (Figure 3a). Based on the polygon diagram obtained on the oil percentage trait, Option500, Sarigol, Modena, Zarfam, Sunday, and Licord genotypes were identified as desirable genotypes due to their greater distance from the origin of the diagram. The environments in this diagram were also divided into four omega environments. The first mega environment, including the BIRJAND1 environment, the second mega environment, the KASHMAR1 environment, the third mega environment, the SHIRAZ1 environment, and other environments were placed in the fourth mega environment. Based on this diagram, Hyola308, Hyola401, and Option500 genotypes in KARAJ1, SANANDAJ1, KARAJ2, BIRJAND2, KASHMAR2, SHIRAZ2, and SANANDAJ2 environments, and Licord genotype in KASHMAR1 environment had the highest level of performance (Figure 3b).

The results showed that all mega environments exist among the test environments, but these mega environments cannot be separated from each other. Sayar and Han (2016) reported even two growing seasons can make a significant difference in yield, allowing the cultivars to be located in several different mega environments. It was also shown that the interaction is positive when the genotypes and environments are in the same sections. While the interaction is negative, when in opposite sections. If they are in an adjacent area, the interaction is more complex. On the other hand, genotypes are close to each other on the map, they may seem efficient in almost all environments, even if the genotypes are far apart, reacting differently from the environment. By using two main axes, this analysis constitutes an advanced understanding of GEI (Kendal et al., 2019). Another same research was accomplished on the stability of rapeseed yield in different regions. Shojaei et al. also selected the Karaj region as the superior environment (Shojaei et al., 2011).

**FIGURE 4:**
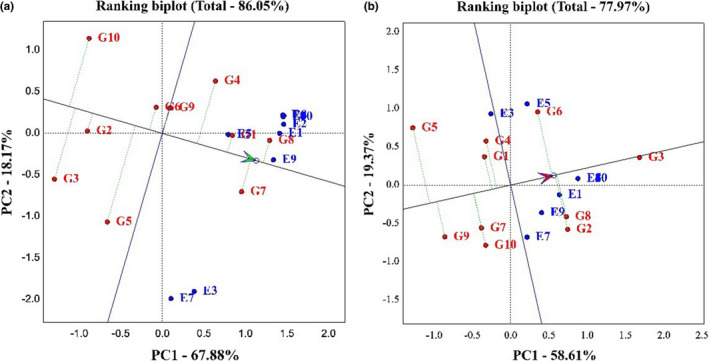
(a) The average environment coordination (AEC) view for the evaluation of 10 canola cultivars in 10 environments with simultaneous base grain yield and yield stability. (b) The average environment coordination (AEC) view for the evaluation of 10 canola cultivars in 10 environments with simultaneous base oil percentage and oil stability.

Figure 4 allows us to determine which genotype has the highest yield, which has the most stability, and which has the highest yield and stability compared to other genotypes. A narrow black line drawn from the negative part of the graph to the positive part of the graph indicates the performance of genotypes in different environments. In other words, any genotype that is on the positive side of this line is identified as a high‐yield genotype. Also, in each section, any genotype that has the least distance from this line is identified as a stable genotype (Rocha et al., 2020). Based on the obtained graph on grain yield, PC1, 67.88% and PC2, 18.17%, and 86.05% of the total variance of the data were explained. Hyola401, Okapi, and Sarigol genotypes were identified as high‐yielding genotypes, and Modena, Option500, and Hyola308 genotypes were identified as low‐yielding genotypes. Also, Okapi, Hyola401, Sarigol, Zarfam, Licord, and Hyola308 genotypes were identified as stable genotypes, and Sunday, Option500, and Modena genotypes as unstable genotypes. In general, because Hyola401, Okapi, and Sarigol genotypes were higher in yield and stability than other genotypes, they were identified as superior cultivars (Figure 4a). According to the oil trait diagram, the first component explained 58.61%, the second component 19.37%, and 77.97% of the total variance of the data. Option500, Hyola401, Hyola308, and Licord genotypes were identified as high‐yielding genotypes, and Sunday and Zarfam genotypes as low‐yielding genotypes. Also, Option500 and Sarigol genotypes were identified as stable genotypes and Sunday genotypes as unstable genotypes (Figure 4b). Several researchers have utilized the mentioned type of chart to identify desirable genotypes in their experiments (Omrani et al., 2022; Shojaei et al., 2011, 2022).

**FIGURE 5:**
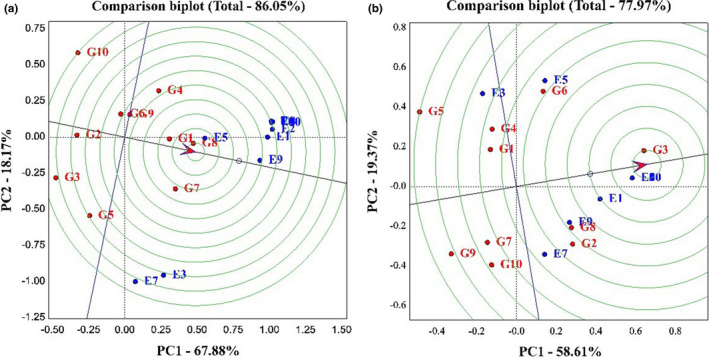
(a) Genotype plus genotype environment interaction (GGE) bi‐plot for comparison of the rapeseed with the ideal genotype based on grain yield and stability. (b) GGE bi‐plot for comparison of the rapeseed with the ideal genotype based on oil percentage and stability.

Figure 5 shows the ranking of genotypes based on the ideal genotype. In this figure, the best point is the center of the concentric circle, which is marked with an arrow. Genotypes with the shortest distance from this arrow are identified as superior genotypes (Oliveira et al., 2018). Based on the graph obtained on grain yield, the first component was 67.88%, the second component was 18.17%, and the total variance was 86.05%. According to this chart, the order of genotypes from the best genotype to the most unfavorable genotype is as follows:

Hyola401 > Sarigol > Okapi > Opera > Zarfam > Licord > Sunday > Hyola308 > Option500 > Modena.

So, the Hyola401 genotype was identified as the best genotype and the Modena genotype as the most undesirable genotype (Figure 5a).

The oil trait graph demonstrates that the first component was 58.61% and the second component was 19.37%, and overall, 77.97% of the total variance of the data was justified. According to this chart, the ranking of genotypes from the best genotype to the most unfavorable genotype is as follows:

Option500 > Hyola401 > Hyola 308 > Licord > Opera > Sarigol > Okapi > Modena > Zarfam > Sunday.

Option500 and Hyola401 genotypes were identified as favorable genotypes and Sunday genotypes as undesirable genotypes.

**FIGURE 6:**
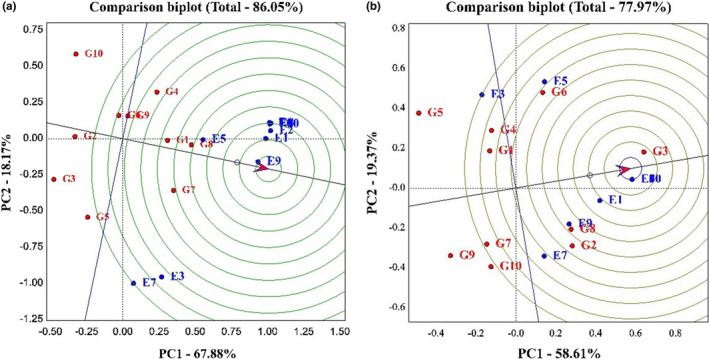
(a) Genotype plus genotype environment interaction (GGE) bi‐plot for comparison of the rapeseed with the ideal environment based on grain yield and stability. (b) GGE bi‐plot for comparison of the rapeseed with the ideal environment based on oil percentage and stability. (G1: Sarigol, G2: Hyola308, G3: Option500, G4: Opera, G5: Sunday, G6: Licord, G7: Okapi, G8: Hyola401, G9: Zarfam, G10: Modena)

Figure 6 shows the ranking of environments based on the ideal environment. In this diagram, the best point is the center of the concentric circle marked with an arrow. Environments located at a shorter distance from this arrow are identified as superior environments (Todd et al., 2018) Based on this graph, in the study of grain yield, the first component explained 67.88% of the data variance, the second component has 18.17%, and 86.05% of the total variance of the data. Based on this chart, the ranking of environments is as follows.

SANANDAJ1 > KARAJ1 > KARAJ2 > BIRJAND2 > KASHMAR2 > SHIRAZ2 > SANANDAJ2 > KASHMAR1 > BIRJAND1 > SHIRAZ1.

Subsequently, the SANANDAJ1 environment was identified with a high screening and differentiation power region, and the SHIRAZ1 as an environment with low differentiation and screening power (Figure 5a).

The data obtained from Figure 5 show that on the oil percentage, the first component was 58.61%, the second component was 19.37%, and 77.97% of the total variance of the data was justified. The ranking of environments based on this chart is as follows:

KARAJ2 > BIRJAND2 > KASHMAR2 > SHIRAZ2 > SANANDAJ2 > KARAJ1 > SANANDAJ1 > KASHMAR1 > SHIRAZ1 > BIRJAND1.

### 3.2 Comparison of AMMI and GGE bi‐plot methods

The results have shown a high correlation between AMMI and GGE bi‐plot methods. According to the AMMI model, more than 96% of the grain yield and more than 94% of the total variance of the data were justified in the oil yield trait. In addition, the GGE bi‐plot method represented more than 86% of the grain yield in the study of traits and approximately about 80% of the variance of the data in the study of oil traits. This high and significant rate of variance percentage justification in both methods indicates the correlation and overlap of two models, AMMI and GGE bi‐plot. Neisse et al. (2018) reported high and close proximity of justified variance in both models. They also concluded in an experiment comparing the AMMI and GGE bi‐plot methods that the two models overlap and complement each other. These two statistical analyses (AMMI and GGE) are more relevant to agricultural researchers because they relate to each two‐way data matrix, and such data emerge from many experiments (Naroui et al., 2013).

## 4 CONCLUSIONS

In this study, a multivariant analysis was conducted to quantify the most stable and compatible cultivars. Indeed, the AMMI and GGE bi‐plot analysis was performed on the studied traits. Based on the AMMI method, it was observed that in terms of grain yield and oil content, the interaction between genotype and environment could play a significant role. To stipulate the degree of importance of the proposed study, an experimental case study was developed. The results depicted the following facts regarding the considered dataset.

The “Zarfam” genotype in grain yield and the “Modena” and “Licord” genotypes in oil percentage had the highest desirability and stability.

The KARAJ1 and SANANDAJ1 regions had high screening power in terms of grain yield and the “KASHMAR2” region in terms of oil percentage.

The GGE bi‐plot method also showed that Hyola401, Okapi, and Sarigol genotypes were more desirable in grain yield and Option500, Hyola401, Hyola308, and Licord genotypes were more desirable in oil content.

In the grain yield trait, Hyola401 genotype, and for the oil percentage trait, Option500 genotype, were identified as the ideal genotypes.

The “SANANDAJ1” environment in grain yield and the “KARAJ2” environment in oil percentage were also identified as ideal environments.

Indeed, by examining the two methods AMMI and GGE bi‐plot, it can be concluded that these two methods are not superior to each other and can complement each other. Therefore, as a general recommendation for multivariant analysis, there is a need for simultaneous consideration of AMMI and GGE bi‐plot techniques to select the most stable and compatible cultivars based on the multivariant properties.

## Data Availability

Data available on request from the authors The data that support the findings of this study are available from the corresponding author upon reasonable request.
